# Inequality in Forensic Medicine in India: The Current Scenario and Way Forward

**DOI:** 10.7759/cureus.62419

**Published:** 2024-06-15

**Authors:** Karthi Vignesh Raj K, Ramdas Ransing, Satyaranjan Sethi, Nilesh Devraj

**Affiliations:** 1 Forensic Medicine, All India Institute of Medical Sciences, Guwahati, Guwahati, IND; 2 Psychiatry, All India Institute of Medical Sciences, Guwahati, Guwahati, IND; 3 Physical Medicine and Rehabilitation, All India Institute of Medical Sciences, Guwahati, Guwahati, IND

**Keywords:** forensic medicine postgraduate, union territories, deficit, india, education, national crime record bureau, trainee index, national medical commission

## Abstract

Background

Forensic Medicine (FM) is one of the core specialties of medicine in India, which serves as a bridge between medical science and India’s justice delivery systems. Although FM experts play a crucial role in handling medicolegal cases, there is an increasing deficit of FM experts in India. This may be due to the inadequate postgraduate (PG) seats across the states and the low uptake of PG seats in FM. This study was conducted to explore the current status of PG seats along with the Forensic Medicine Toxicology (FMT)/FM index across Indian states.

Methodology

In this cross-sectional study, data on the number of PGs in FMT/FM and the institutes offering PG courses in FMT/FM were searched on the official website of the National Medical Commission. The data available on the website until November 2023 were incorporated. The FMT/FM index was calculated at the national and state levels, and the states were graded based on the national FMT/FM index.

Results

The national FMT/FM PG trainee index was 0.054. Considering this as the reference value, grading of the FMT/FM PG trainee index was done as the highest (1.159 to 0.308), higher than the nation (0.054 but less than 0.308), lower than the nation (0.054 but higher than 0.00), and zero. Among all the states and union territories, Andaman & Nicobar, Arunachal Pradesh, Dadra and Nagar Haveli, Jammu & Kashmir, Lakshadweep, Mizoram, Nagaland, Sikkim, and Ladakh had zero FMT/FM PG index due to non-availability of any PG seats for FMT/FM. In total, 20 states had an FMT/FM PG index higher than India’s FMT/FM PG index headed by Pondicherry (1.159), followed by Chandigarh (0.429) and Goa (0.308).

Conclusions

PG seats were highly deficient in several states, which is more likely to affect justice delivery in the future across these states. This study has a few limitations, as we did not explore the actual intake of these PG seats across different states. We anticipate a lower intake of PG seats due to factors such as low demand, fewer job opportunities, and monetary gain. However, this needs further exploration in future studies.

## Introduction

India is one of the most populous countries in the world. Hence, it is the responsibility of the Indian government to take care of its citizens’ “health” which includes a state of complete physical, mental, and social well-being, as stated by the World Health Organization (WHO). A doctor-patient ratio of 1:1,000, i.e., “the number of physicians available per every 10,000 inhabitants in a population, at a given year, for a given country, territory, or geographic area” is the target set by the WHO [1]. To achieve this target, the Indian government took a firm decision a few years ago to open new medical colleges all over the country to improve the healthcare system by increasing the number of doctors. This resulted in increasing the undergraduate (UG) seats to 79% and postgraduate (PG) seats to almost 93%, thereby achieving a doctor-patient ratio of 1:834 which is significantly better than expected by the WHO [2,3].

The National Medical Commission (NMC) controls the recognition of medical colleges (government and private), UG and PG courses, and faculties all over India [4] except for institutes of national importance such as the All India Institute of Medical Sciences (AIIMS), Post Graduate Institute of Medical Examination and Research (PGIMER), National Institute of Mental Health and Neurosciences (NIMHANS), and Jawaharlal Institute of Postgraduate Medical Education and Research (JIPMER). During the tenure, a UG is trained to deal with various medicolegal cases. To build expertise in the field they require postgraduation in Forensic Medicine (FM). FM is a branch of medicine that deals with the administration of medical and paramedical knowledge to aid in the administration of justice [5]. The opinions of FM experts come into the picture whenever an investigating agency or judiciary requires a medical opinion to adjudicate their opinion on violent crimes. According to the National Crime Record Bureau (NCRB) report published in 2021, a total of 3,663,360 cognizable crimes were registered throughout India, of which 1,100,425 constituted offenses against the human body, accounting for 30% of total IPC crimes. On the other hand, cases of hurt constituted 585,774 (53.2%), followed by cases of causing death by negligence at 146,195 (13.3%). There is an overall increase in offenses from 77.4% in 2020 to 80.5% in 2021 against the human body, i.e., an increase of 5.1% [6]. This highlights the importance and requirement of an FM specialist to aid in coming to the conclusion by the judiciary in such offenses committed against the human body.

This study aims to identify discrepancies to highlight the requirement of FM experts or to obtain the data of doctors trained in the FM field currently present all over the country. To our knowledge, to date, no study has established the data related to the existing/sanctioned FM specialists which could be considered essential given the increased/prevailing crime rates. The present study highlights the deficit and distribution of FM PG trainee seats/institutes and estimates the Forensic Medicine Toxicology/Forensic Medicine Postgraduate Trainee Seats Index (FMT/FM-PGTI) among the Indian states and union territories (UTs).

This article was previously posted as a preprint on Research Square under the DOI 10.21203/rs.3.rs-3814976/v1.

## Materials and methods

**Figure 1:**
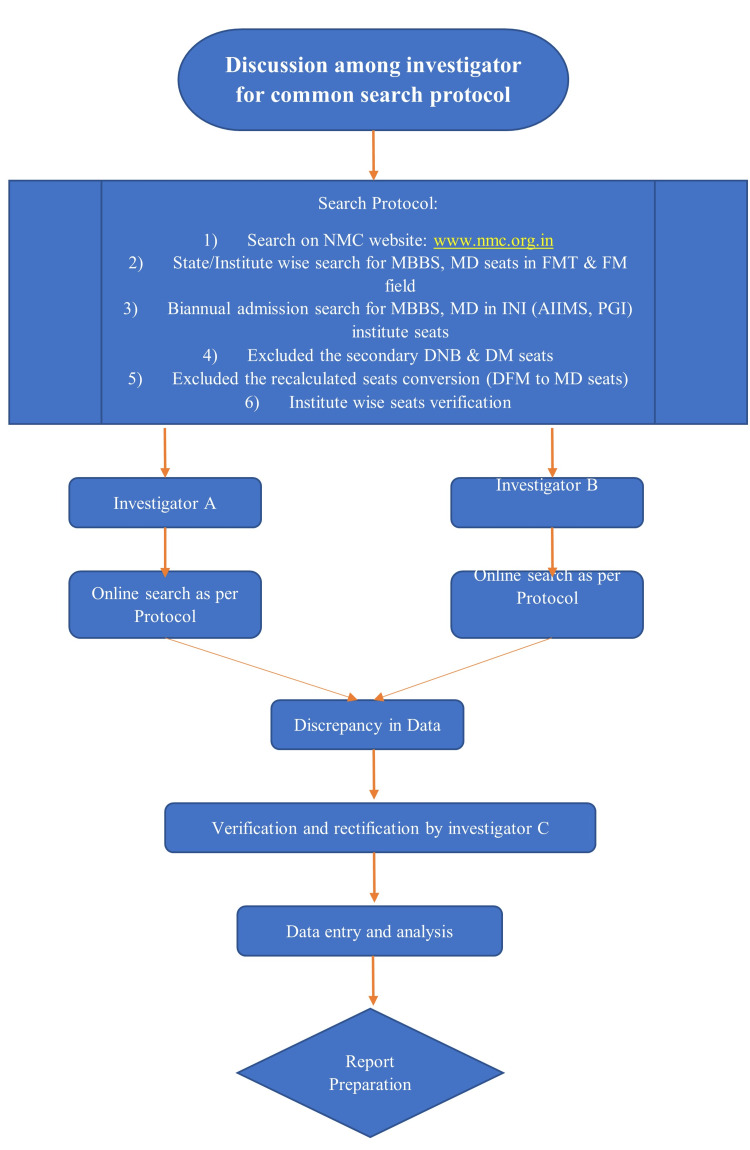
Investigation flowchart. NMC = National Medical Commission; MD = Doctor of Medicine; DM = Doctorate of Medicine; DNB = Diplomate of National Board; DFM = Diploma in Forensic Medicine; INI = Institutes of National Importance; FM = Forensic Medicine; FMT = Forensic Medicine Toxicology

This cross-sectional study was conducted between September 1, 2023, and November 30, 2023. The official website of the National Medical Commission (NMC) was utilized to retrieve the data regarding sanctioned PG seats in FM and FMT according to state and institute. Any PG degree in the medical field should be recognized or permitted by NMC to practice in India. Non-approved courses were excluded from this study. All institutes, i.e., government, semi-government, and private, where PG trainee seats are available were included. Two independent investigators conducted an online search for FMT and FM PG trainee seats and institutes (Figure 1).

The two investigators collected data separately which was cross-verified by a third investigator for any discrepancy and rectification. FMT/FM-PGTI was calculated for the state (FMT/FM PGTI for state = number of Forensic Medicine trainee seats per year × 100,000/state population) and for the nation (FMT/FM PGTI for nation = number of Forensic Medicine trainee seats per year × 100,000/nation population) using the formula with population data (June 2023) provided by the Unique Identification Authority of India [8]. Ethical permission to conduct this study was not required as data were available in the public domain. We consolidated the data for publication and portrayed the exact current scenario.

## Results

In India, there are 706 MBBS teaching institutes during the mentioned study period, including government, semi-government, commercial, and trust-run institutes. Each year, 108,840 seats are made available for MBBS admission through these institutes. India has a total of 37 states including UTs, of which 28 states provide MBBS courses and 751 trainee seats for the PG course of FMT/FM. This amounts to 0.69% of the total number of MBBS seats available throughout the country (Table 1).

**Table 1:** Distribution of MBBS, postgraduation FMT/FM training institutes, trainee seats, population, and FMT/FM postgraduate trainee index across Indian states and union territories. FMT/FM = Forensic Medicine Toxicology/Forensic Medicine

States	Total number of MBBS training institutes		Number of postgraduate FMT training institutes					Postgraduate FMT trainee seats					Population as per UIDAI (2022)	FMTI index (%)
			Forensic Medicine and Toxicology		Forensic Medicine		Total (n)	Forensic Medicine and Toxicology		Forensic Medicine		Total (n)		
	Government and private institutes (n)	UG seats	Government (n)	Private (n)	Government (n)	Private (n)		Government (n)	Private (n)	Government (n)	Private (n)			
Andaman & Nicobar	1	114	0	0	0	0	0	0	0	0	0	0	388,388	0
Andra Pradesh	37	6,485	6	3	5	0	14	27	5	15	0	47	51,874,942	0.090603
Arunachal Pradesh	1	50	0	0	0	0	0	0	0	0	0	0	1,249,327	0
Assam	13	1,550	4	0	0	0	4	10	0	0	0	10	30,768,862	0.0325
Bihar	21	2,765	6	0	0	0	6	16	0	0	0	16	109,664,801	0.01459
Chandigarh	1	150	2	0	0	0	2	5	0	0	0	5	1,163,469	0.429749
Chattisgarh	14	2,005	3	1	2	0	6	7	2	8	0	17	28,295,179	0.060081
Dadra & Nagar Haveli	1	177	0	0	0	0	0	0	0	0	0	0	601,398	0
Daman & Diu	0	0	0	0	0	0	0	0	0	0	0	0		
Delhi	10	1,497	5	0	0	0	5	37	0	0	0	37	22,759,589	0.162569
Goa	1	180	1	0	0	0	1	5	0	0	0	5	1,620,390	0.308568
Gujrat	40	7,150	8	3	0	1	12	31	5	0	3	39	65,441,370	0.059595
Haryana	15	2,185	3	3	2	0	8	12	7	4	0	23	30,164,370	0.076249
Himachal Pradesh	8	920	2	1	0	0	3	3	3	0	0	6	7,758,034	0.077339
Jammu & Kashmir	12	1,339	0	0	0	0	0	0	0	0	0	0	11,734,709	0
Jharkhand	9	980	1	0	0	0	1	6	0	0	0	6	35,862,550	0.016731
Karnataka	70	11,745	10	24	3	3	40	22	62	11	9	104	64,849,050	0.27824
Kerala	33	4,655	5	1	1	0	7	13	2	2	0	17	37,377,817	0.045482
Lakshadweep	0	0	0	0	0	0	0	0	0	0	0	0	74,087	0
Madhya Pradesh	27	4,800	5	2	3	1	11	24	6	8	3	41	78,068,045	0.052518
Maharashtra	68	10,845	18	4	3	1	26	61	7	11	3	82	118,776,600	0.069037
Manipur	4	525	2	0	0	0	2	5	0	0	0	5	2,637,796	0.189552
Meghalaya	1	50	1	0	0	0	1	3	0	0	0	3	2,401,534	0.12492
Mizoram	1	50	0	0	0	0	0	0	0	0	0	0	1,198,300	0
Nagaland	1	100	0	0	0	0	0	0	0	0	0	0	1,367,447	0
Orisa	17	2,525	4	3	0	0	7	16	9	0	0	25	44,020,860	0.056791
Pondicherry	9	1,830	1	2	1	1	5	3	6	4	2	15	1,293,758	1.159413
Punjab	12	1,800	3	0	0	0	3	10	0	0	0	10	31,240,741	0.032009
Rajasthan	35	5,575	7	4	1	1	13	39	7	4	3	53	75,436,152	0.070258
Sikkim	1	150	0	0	0	0	0	0	0	0	0	0	576,988	0
Tamilnadu	74	11,600	10	4	0	1	15	28	11	0	5	44	74,224,381	0.05928
Telangana	56	8,490	5	1	3	1	10	26	2	9	2	39	38,523,838	0.101236
Tripura	2	225	1	0	0	0	1	3	0	0	0	3	3,826,432	0.078402
Uttarakhand	8	1,150	2	1	2	0	5	27	2	6	0	35	11,616,082	0.301306
Uttar Pradesh	68	9,903	6	7	1	0	14	27	13	3	0	43	2,18,447,362	0.019684
West Bengal	35	5,275	6	0	2	0	8	19	0	2	0	21	96,492,565	0.021763
Ladakh	0	0	0	0	0	0	0	0	0	0	0	0	240,321	0
Total	706	108,840	127	64	29	10	230	485	149	87	30	751	1,302,037,534	0.057679

As shown in Table 2, 19 states with 10 or less than 10 to 0 medical institutes that offer MBBS courses, with three UTs (Daman & Diu, Lakshadweep, and Ladakh), are devoid of any medical institutes. Four states and two UTs (Andaman & Nicobar, Arunachal Pradesh, Dadra & Nagar Haveli, Mizoram, Nagaland, and Sikkim) have medical institutes offering MBBS courses but no PG courses in FMT or FM. Seven states and three UTs (Chandigarh, Delhi, Goa, Himachal Pradesh, Jharkhand, Manipur, Meghalaya, Pondicherry, Tripura, and Uttarakhand) offer MBBS courses along with PG courses in FMT or FM.

**Table 2:** Distribution of 19 states with 10 or fewer than 10 to 0 MBBS medical institutes, postgraduation FMT/FM training institutes, trainee seats, population, and FMT/FM postgraduate trainee index across Indian states and union territories. FMT/FM = Forensic Medicine Toxicology/Forensic Medicine

States	Total number of MBBS training institutes		Number of postgraduate FMT training institutes					Postgraduate FMT trainee seats					Population as per UIDAI (2022)	FMTI index (%)
			Forensic Medicine & Toxicology		Forensic Medicine		Total (n)	Forensic Medicine & Toxicology		Forensic Medicine		Total n		
	Government and private institute (n)	UG seats	Government (n)	Private (n)	Government (n)	Private (n)		Government (n)	Private (n)	Government (n)	Private (n)			
Andaman & Nicobar	1	114	0	0	0	0	0	0	0	0	0	0	388,388	0
Arunachal Pradesh	1	50	0	0	0	0	0	0	0	0	0	0	1,249,327	0
Chandigarh	1	150	2	0	0	0	2	5	0	0	0	5	1,163,469	0.429749
Dadra & Nagar Haveli	1	177	0	0	0	0	0	0	0	0	0	0	601,398	0
Daman & Diu	0	0	0	0	0	0	0	0	0	0	0	0		
Delhi	10	1,497	5	0	0	0	5	37	0	0	0	37	22,759,589	0.162569
Goa	1	180	1	0	0	0	1	5	0	0	0	5	1,620,390	0.308568
Himachal Pradesh	8	920	2	1	0	0	3	3	3	0	0	6	7,758,034	0.077339
Jharkhand	9	980	1	0	0	0	1	6	0	0	0	6	35,862,550	0.016731
Lakshadweep	0	0	0	0	0	0	0	0	0	0	0	0	74,087	0
Manipur	4	525	2	0	0	0	2	5	0	0	0	5	2,637,796	0.189552
Meghalaya	1	50	1	0	0	0	1	3	0	0	0	3	2,401,534	0.12492
Mizoram	1	50	0	0	0	0	0	0	0	0	0	0	1,198,300	0
Nagaland	1	100	0	0	0	0	0	0	0	0	0	0	1,367,447	0
Pondicherry	9	1,830	1	2	1	1	5	3	6	4	2	15	1,293,758	1.159413
Sikkim	1	150	0	0	0	0	0	0	0	0	0	0	576,988	0
Tripura	2	225	1	0	0	0	1	3	0	0	0	3	3,826,432	0.078402
Uttarakhand	8	1,150	2	1	2	0	5	27	2	6	0	35	11,616,082	0.301306
Ladakh	0	0	0	0	0	0	0	0	0	0	0	0	240,321	0

There are 230 PG teaching institutions in India (156 government and 74 private) that provide PG courses in FMT or FM. Of these 751 seats available for applicants who desire to pursue careers in FMT or FM, 572 (76.16%) are in government-run institutions and 179 (23.83%) are in private institutions. Among all the states in India, Karnataka has the highest number of seats (104 seats; 33 in government and 71 in private), followed by Maharashtra (82 seats; 72 in government and 10 in private), and Rajasthan (53 seats; 42 in government and 11 in private). Meghalaya and Tripura states provide three seats, followed by Chandigarh, Manipur, and Goa with five PG seats each in government institutes.

**Figure 2:**
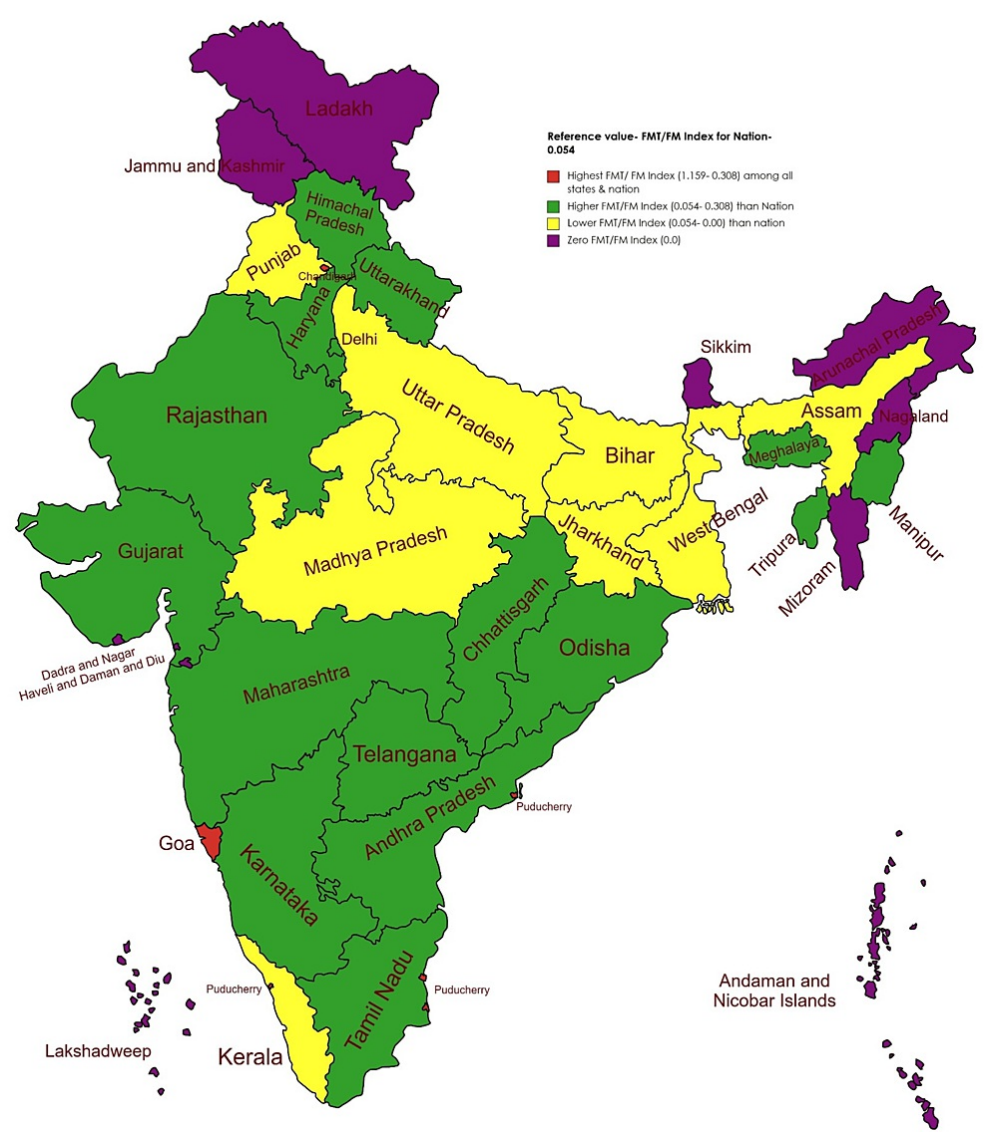
Indian map showing the FMT/FM index for the nation and states. Image credit: Dr. N A Devraj, Associate Professor, Department of Forensic Medicine & Toxicology, All India Institute of Medical Sciences, Guwahati, India. FMT/FM = Forensic Medicine Toxicology/Forensic Medicine

FMT/FM-PGTI for the nation at 0.054 was calculated according to Table 3. The individual state FMT/FM-PGTI was calculated. By considering the national FMT/FM-PGTI as a reference value, the grading of the state FMT/FM-PGTI was done, as depicted in Figure 2 and Table 3.

**Table 3:** FMT/FM PG index for the nation and state. FMT/FM = Forensic Medicine Toxicology/Forensic Medicine; PG = postgraduate

Grade	FMT/FM index	States with FMT/FM index
Highest	1.159–0.308	Pondicherry (1.159) tops, followed by Chandigarh (0.429) and Goa (0.308)
Higher	>0.054 but <0.308	Andhra Pradesh (0.0906), Chhattisgarh (0.06008), Delhi (0.016), Gujrat (0.0596), Haryana (0.07625), Himachal Pradesh (0.07734), Karnataka (0.27824), Maharashtra (0.06904), Manipur (0.018), Meghalaya (0.1249), Orissa (0.05679), Rajasthan (0.07026), Tamil Nadu (0.05928), Telangana (0.10124), Tripura (0.0784), and Uttarakhand (0.301)
Reference value	0.054	India
Lower <0.054	0.054–0.00	Assam (0.032), Bihar (0.014), Jharkhand (0.016), Kerala (0.045), Madhya Pradesh (0.052), Punjab (0.032), Uttar Pradesh (0.019), and West Bengal (0.021)
Zero	0.00	Andaman & Nicobar, Arunachal Pradesh, Dadra & Nagar Haveli, Jammu & Kashmir, Lakshadweep, Mizoram, Nagaland, Sikkim, and Ladakh

Among all the states & UTs, Andaman & Nicobar, Arunachal Pradesh, Dadra & Nagar Haveli, Jammu & Kashmir, Lakshadweep, Mizoram, Nagaland, Sikkim, and Ladakh have zero FMT/FM-PGTI due to non-availability of any PG seats for FMT/FM. Other states such as Assam (0.032), Bihar (0.014), Jharkhand (0.016), Kerala (0.045), Madhya Pradesh (0.052), Punjab (0.032), Uttar Pradesh (0.019), and West Bengal (0.021) have FMT/FM-PGTI less than India’s FMT/FM-PGTI. Apart from all the above states, 20 states have FMT/FM-PGTI higher than India’s FMT/FM-PGTI, headed by Pondicherry (1.159), followed by Chandigarh (0.429) and Goa (0.308). The FMT/FM-PGTI for India’s densely populous states of Uttar Pradesh, Maharashtra, Bihar, and West Bengal are 0.019, 0.069, 0.014, and 0.02176, respectively.

## Discussion

The health education system in India has been growing exponentially in the last few decades [9]. Previously, medical institutes in India were regulated by the Medical Council of India which is now replaced by NMC [10-12]. According to the NMC, MBBS Admission Regulation 2020, the faculty strength required for FMT at the time of recognition of the first batch of 100 MBBS students is five (professor, one; associate professor, one; assistant professor, one; and tutor/demonstrator/senior resident, two) [13]. The ratio of PG teachers to PG students admission is professor three students, associate professor two students (government or non-government-established institute with more than 15 years), or one student [14]. The total PG trainee student intake depends upon the number of professors and associate professors in the department. However, the mentioned faculty strength for 150, 200, and 250 MBBS students continues to remain the same despite NMC having sanctioned additional faculty such as assistant professors and tutors/demonstrators/senior residents who are not eligible to take PG students under them (Table 4).

**Table 4:** The FMT/FM faculty strength for MBBS admission. Minimum FMT/FM faculty strength required for MBBS admission as per NMC Gazette: Minimum Requirements for Annual MBBS Admissions Regulation, 2023, dated 16/08/2023 [15]. FMT/FM = Forensic Medicine Toxicology/Forensic Medicine; NMC = National Medical Commission

MBBS admission capacity	Professor	Associate professor	Assistant professor	Tutor/Demonstrator/Senior resident
100	01	01	01	02
150	01	01	01	03
200	01	01	02	04
250	01	01	04	04

This highlights that the intake of PG trainees in FMT/FM depends upon the number of existing professors and associate professors in FMT departments. The second reason could be the lack of sufficient faculty eligible to guide PG trainees [16].

Currently, FMT/FM trainees have fewer professional options, particularly in government or private medical colleges, the health system, or the private sector. The low employment for FM experts in any healthcare facility promotes lawsuits [17] which eventually increase day by day. This could be a demotivating aspect for choosing this sector as a career path compared to other fields with extremely strong options aside from work.

The handling of medicolegal work marked its beginning in the British era. Earlier, medicolegal work was assigned to civil surgeons due to a lack of experts trained in FMT/FM [18]. The conduct of autopsy plays a significant role in FM, along with conducting various types of research which could lead to improving the existing clinical practices. An example is the COVID-19 pandemic witnessed by the entire world. Not only clinicians but the general public and government officials were interested in understanding the pathological changes in the human body due to COVID-19 infection. An autopsy has such an implication for the medical field which is considered to be the least in the aspects of funding and allocation of resources [19]. The FMT/FM trainee is the future FM expert who plays an imminent role in the process of collecting vital evidence from the body which plays a major role in assisting in delivering justice. Furthermore, in cases related to allegations against the exploitation or violation of human rights, the biomedical evidence collected by an FMT expert/trainee helps correlate and prove or disprove the allegation raised against the state evidence [20-23]. The impact of the lack of an FMT/FM trainee or future FMT/FM experts in the state with a low FMT/FM-PGTI will be highlighted during the investigation of high-profile criminal or human rights violation cases.

Although the law says that any registered medical practitioner is eligible to conduct an autopsy [24], the reality is that not all registered medical practitioners are well-trained or have the knowledge to conduct an autopsy without a flaw. An autopsy is a vital procedure that guides investigative authorities based on medical facts that are perceived by a trained professional. The training is vital because reports such as RMP dispatches play a role in the judgments delivered either to save or to punish a human being. Hence, through this study, we tried to reinforce the existing demand for experts in the field of FM for smooth medicolegal practice.

Strengths and limitations

This is the first study to showcase the current scenario of the nation and states on FMT/FM-PGTI. This study has limitations, particularly data on PGs from newer institutes of national importance such as AIIMS, which are not available on the NMC website.

Recommendations

Corrective actions must be implemented such as a revamp of the FMT curriculum, departmental faculty strength, and an approach of interdepartmental, intersectoral superspecialty in the topic to obtain newer work opportunities.

## Conclusions

Low FMT/FM-PGTI may be due to fewer faculty in the department, as well as the lower appeal of FM in terms of demand, saturation in the field, job potential, and monetary reward in contrast to other specialties. To address this, the department’s faculty strength must be revised, and FMT/FM trainees must be trained using an interdepartmental and intersectoral approach, as well as a revamp of the FM curriculum. Along with this, it is necessary to create separate faculty strengths at various levels of health department services. Development of a superspecialty branch needs to be done for newer job opportunities in any sector to attract graduates.
